# Epigenetic clocks: advancing biological age measures towards meaningful clinical use

**DOI:** 10.1016/j.ebiom.2026.106175

**Published:** 2026-02-11

**Authors:** eBioMedicine

The distinction between chronological and biological age has become central to modern ageing research. Although chronological age simply counts time, biological age aims to capture the molecular, physiological, and environmental changes that shape frailty and disease vulnerability. Public interest in biological age has grown rapidly; yet, important questions remain about the reliability of these measures in commercial wellness settings and how they should be used in clinical practice. Measuring such a complex parameter poses substantial challenges. Clarifying how these factors influence disease risk, and whether they can be meaningfully integrated into a robust biomarker, is essential for advancing the science of ageing and supporting the clinical translation of biological age measures.

Ageing clocks have become influential tools in geroscience and longevity research, evolving from early statistical models that predicted chronological age to more sophisticated machine-learning systems that integrate omics, clinical biomarkers, and lifestyle factors. The most widely used tools rely on DNA-methylation algorithms known as epigenetic clocks, which quantify methylation changes at short DNA regions rich in cytosine–guanine dinucleotides (CpG islands), and are now widely applied to predict disease risk, evaluate exposures, and track ageing-related physiological decline.

Recently, two studies have shown that modifiable cardiovascular behaviours shape both the level and the rate of biological ageing. A longitudinal multi-cohort study published by eBioMedicine in December, 2025, reported that smoking, higher BMI, elevated glucose, and poor blood pressure profiles accelerate ageing as measured by DNA epigenetic tools such as DunedinPACE, whereas physical activity and healthier diet slow it. The study further reinforces the observation that individuals who move into faster ageing trajectories tend to exhibit poorer cardiovascular profiles over time. Complementary findings in BMC Medicine linked favourable cardiovascular health to lower epigenetic age acceleration, with sex-specific influences: nicotine avoidance and glucose were stronger in male individuals, whereas physical activity, glucose regulation, and healthy BMI were most influential in female individuals.

Beyond cardiometabolic health, the predictive value of biological ageing extends into other organ systems. DNA epigenetic-derived clocks are emerging as useful adjuncts for early risk identification, capturing vascular vulnerability that might not yet be clinically apparent. A synthesis of 13 studies in a November, 2025 systematic review and meta-analysis found that individuals with accelerated biological ageing, as measured by DNA epigenetic clocks, were consistently more likely to have a stroke. The association was stronger for first ever stroke than for recurrent events, suggesting that epigenetic ageing captures vascular vulnerability not fully explained by traditional risk factors.

Most epigenetic clocks still derive their predictive features from correlation rather than causation, leaving open questions about whether methylation changes drive or accompany environmental exposures and organ decline. Efforts to bridge statistical ageing biomarkers with molecular pathways are emerging, including a December, 2025 *eBioMedicine* study on biological ageing and colorectal cancer (CRC) that integrated longitudinal epidemiology, Mendelian randomisation, and methylation quantitative trait locus analyses. The authors identified 15 ageing-linked CpG sites whose methylation appears to influence CRC risk through altered expression of genes such as *TNF, NCF2, BICC1*, and *DIP2B*. Accelerated biological ageing also showed stronger predictive value for early-onset CRC, suggesting that age-related epigenetic drift might confer carcinogenic vulnerability earlier in life.

Although not definitive proof of mechanism, this work highlights the importance of linking ageing biomarkers to disease risk. A recent *npj Aging* commentary, provocatively titled “Do We Actually Need Aging Clocks?”, argued that the defining principles of biological age remain conceptually unresolved. Current clocks capture broad trends but often diverge in their estimates, reflecting differences in population demographics, environmental exposures, lifestyles, disease stages, and tissue-specific methylation patterns. Sex is another important source of biological variation, with several studies showing that male and female individuals exhibit distinct epigenetic ageing trajectories, yet these differences are not consistently reported or modelled. Moreover, most epigenetic clocks are trained on blood or saliva samples, limiting their ability to generalise across tissues. Beyond these biological sources of variation, clocks also differ substantially from one another. Tools such as TEAPEE allow users to explore how different models relate to diverse phenotypes, illustrating how strongly outputs depend on the specific algorithm and training cohort. A December, 2025 *Nature Communications* analysis further highlighted this heterogeneity, reporting substantial variation in effect sizes and in the number of significant associations across 14 clocks and 174 disease outcomes, with no single model emerging as uniformly superior.

As research linking biological ageing to chronic disease accelerates, their potential remains considerable: from clarifying ageing mechanisms and integrating molecular biomarkers into preventive strategies, to serving as more informative endpoints in clinical trials and diagnostics. Several ongoing clinical trials, summarised in a 2023 review, have already begun incorporating ageing clocks as primary or secondary endpoints, reflecting their rapid uptake even without clear evidence that they can reliably track intervention driven biological change. Key mechanistic and methodological gaps must be addressed to ensure these biomarkers mature into clinically meaningful instruments.

